# A Complex Case of COVID-19 Pneumonitis in a Patient With Follicular Lymphoma

**DOI:** 10.7759/cureus.73673

**Published:** 2024-11-14

**Authors:** Ameya Elizabeth Benedict, Graham Hantman, Kavitha Paul, Saeed Akbar

**Affiliations:** 1 Acute Medicine, Ashford and St. Peter's NHS Trust, Surrey, GBR; 2 Respiratory Medicine, Ashford and St. Peter's NHS Trust, Surrey, GBR

**Keywords:** covid-19, cycle threshold, ground-glass opacity, hematologic malignancy, immunocompromised

## Abstract

Protracted COVID-19 is increasingly recognised in immunocompromised patients, particularly those with haematological malignancies. Here, we present the case of a patient with protracted COVID-19 and an underlying B-cell malignancy. Standard COVID-19 treatment with remdesivir and steroids proved ineffective in this patient as she continued to have evolving ground-glass opacities on imaging. A multidisciplinary involvement altered treatment to include a combination of antivirals nirmatrelvir/ritonavir (Paxlovid) and remdesivir, a monoclonal antibody and immunoglobulins leading to a clinical cure. This report highlights the need for a more tailored approach in this patient sub-group than the rest of the population.

## Introduction

Coronavirus disease 2019 (COVID-19), caused by SARS-CoV-2, has a spectrum of features, ranging from asymptomatic to severe, life-threatening symptoms [1]. Immunosuppressed patients are at risk of more severe features and the management is complicated by persistent COVID-19 infection [2]. One of the imaging modalities commonly utilised in diagnosing and prognosticating COVID-19 is CT of the thorax, and the common findings are ground-glass opacifications (GGOs) with or without co-existing consolidation [3]. GGO on CT is defined as increased attenuation of the lung parenchyma wherein the broncho-vascular markings remain intact, and the underlying aetiology is the filling of alveoli with cells or fluid. However, this finding is not specific to viral pneumonitis. In COVID-19 pneumonitis, the acute phase is characterised by peripheral, subpleural GGOs. The differentials encompass other inflammatory/infectious conditions, even cancer, and require careful interpretation in a clinical context, as highlighted by the case below of an immunosuppressed patient [4]. In immunosuppressed patients, other opportunistic infections such as *Pneumocystis jirovecii *pneumonia and cytomegalovirus pneumonia should be considered, which also show GGOs [4]. 

## Case presentation

**Figure 1:**
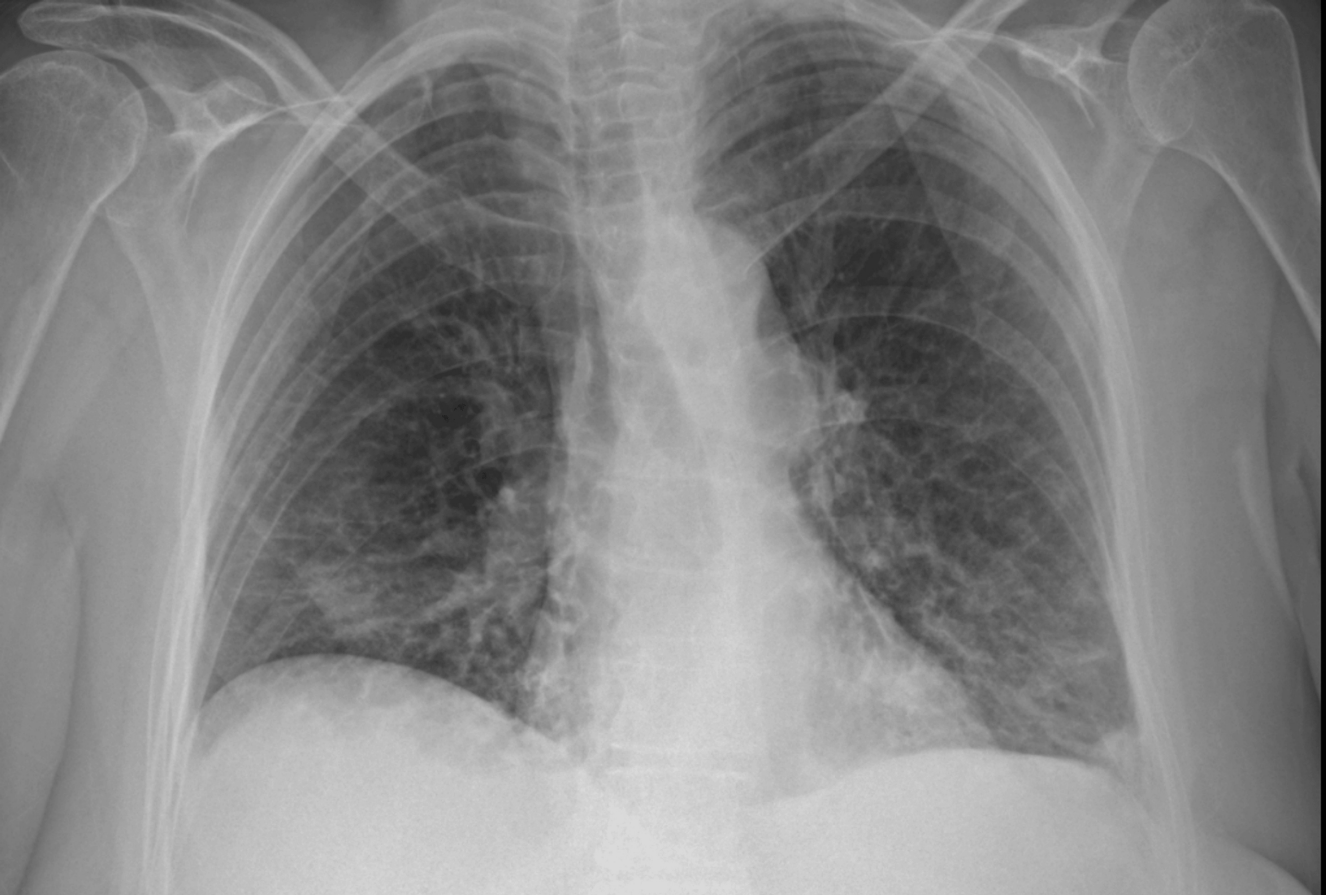
Chest radiograph on day 36 showing bilateral ground-glass changes in the lower hemithorax.

A 72-year-old female presented to the Emergency Department with a 15-day history of cough and breathlessness having tested positive for COVID-19 on lateral flow immunoassay on day 1 of symptoms. She had a history of follicular lymphoma on cyclical immunotherapy obinutuzumab (anti-CD20). There were no radiographical changes on the plain chest radiograph, and she was discharged with oral antibiotics. On day 36, she presented again to the hospital with worsening shortness of breath and hypoxia with bilateral ground glass shadowing on a plain chest radiograph (Figure 1). She was given supplemental oxygen via nasal cannula and steroids and discharged after a week of treatment.

**Figure 2:**
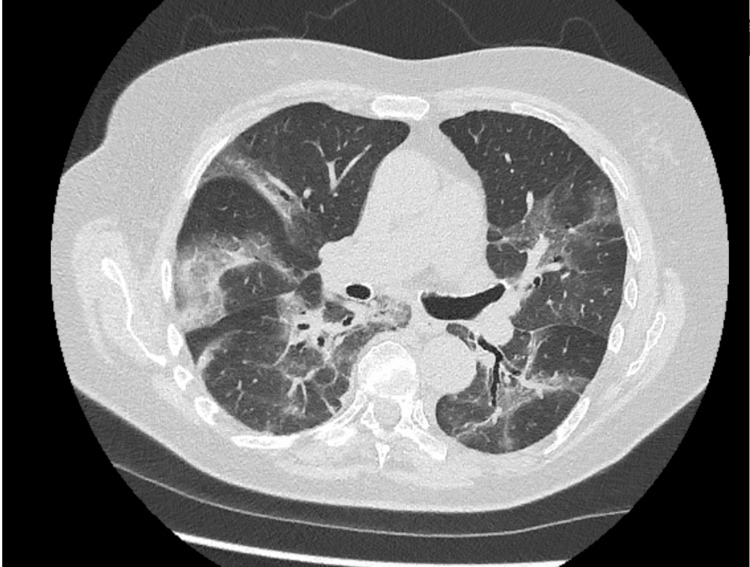
CT of the thorax on day 51 showing evolving ground-glass opacifications.

**Figure 3:**
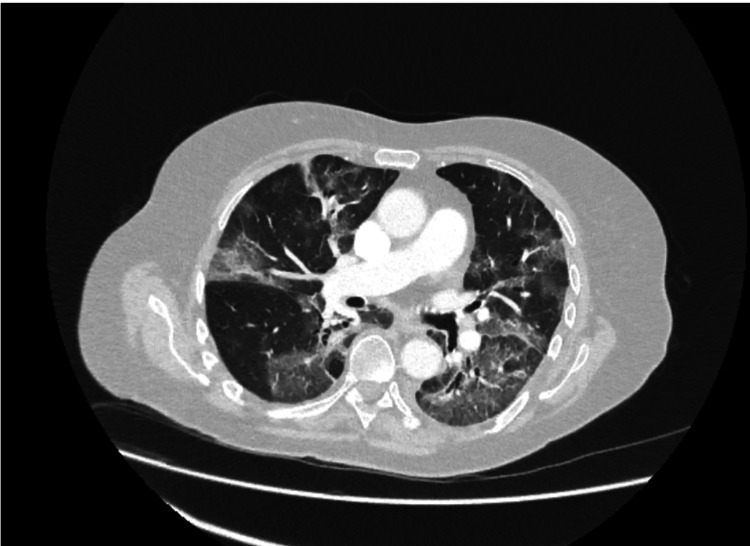
CT of the thorax on day 72 suggestive of Pneumocystis jirovecii infection.

Despite initial treatment, she experienced recurrent exacerbations over two months, culminating in progressive respiratory distress requiring hospital admission and oxygen support. Serial CT of the thorax on day 51 (Figure 2) and day 72 (Figure 3) showed evolving GGOs consistent with COVID-19 pneumonia, indicative of disease severity. Despite treatment with antibiotics, steroids, and remdesivir, her symptoms continued to worsen, prompting investigations for alternative diagnoses. The second CT of the thorax on day 72 (Figure 3) also demonstrated bilateral GGO, suggestive of *Pneumocystis jirovecii* infection, prompting initiation of co-trimoxazole. As the patient was unwell and was on high-flow nasal oxygen, she could not undergo a bronchoalveolar lavage for further testing.

**Figure 4:**
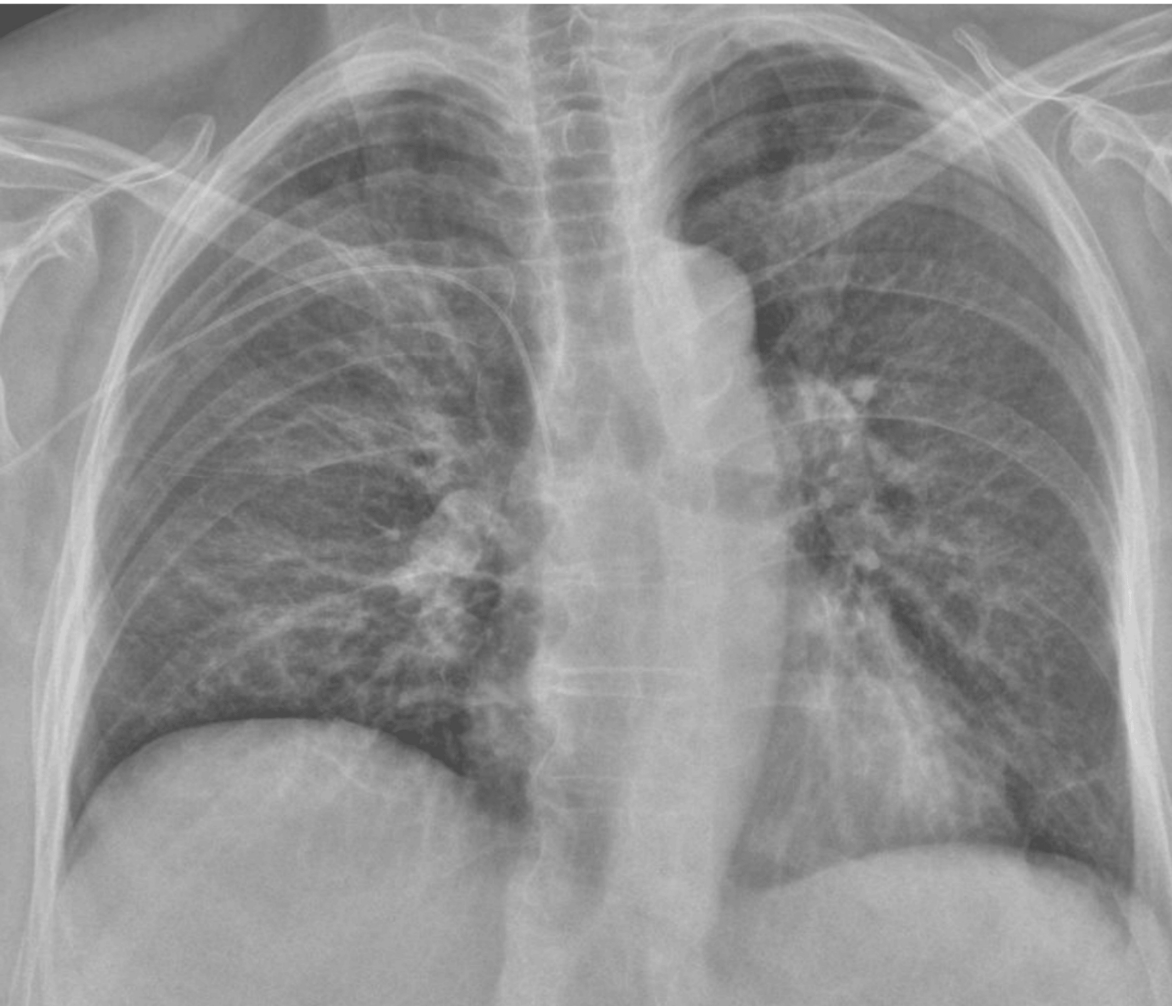
Chest radiograph showing radiological improvement of ground-glass changes after treatment with monoclonal antibody, antivirals, and immunoglobulins.

Further investigation with CT pulmonary angiogram (day 105), including viral load testing and cycle threshold (Ct) counts, favoured a diagnosis of COVID-19 pneumonitis over *Pneumocystis jirovecii* pneumonia. Treatment was modified after discussion with the tertiary virology centre and haematology to include monoclonal antibodies (sotrovimab), antivirals (remdesivir and nirmatrelvir/ritonavir), and immunoglobulin infusion to target viral clearance and mitigate inflammation. This therapeutic combination led to a marked improvement in symptoms and a reduction in oxygen requirements (Figure 4). She was discharged with an immunology clinic follow-up.

## Discussion

Several risk factors have been identified for severe COVID-19, and increasing age is widely recognised [5]. In addition, this patient suffered from a haematological malignancy and was receiving obinutuzumab (anti-CD20), which is linked to prolonged and more severe COVID-19 [6]. This patient was initially managed with corticosteroids with no improvement in her symptoms. As she continued to worsen clinically and radiologically, other differentials including *Pneumocystis jirovecii* pneumonia, a recognised complication of immunosuppression, had to be included and treated. In this patient, cycle threshold values and genetic testing for SARS-CoV-2 aided in diagnosis, and low cycle threshold values indicated a high viral load [7]. A multi-disciplinary approach involving haematology and virology changed the approach towards treatment. A combination of monoclonal antibodies and two antivirals were administered to the patient. There are various reports of patients with lymphoma and other haematological malignancies suffering from severe COVID-19 who have responded to the above combination [8]. Paxlovid, a combination of nirmatrelvir and ritonavir, is an effective agent with a reported lower mortality rate than other anti-viral agents [9].Another outcome of the multidisciplinary discussion was the administration of intravenous immunoglobulins (IVIGs), expediting viral clearance and subsequent clinical improvement. A study by Billi et al. in a population requiring oxygen but not intensive admission, akin to this patient, reported similar results [10]. HM-COV 2.0, a single-centric study by Oliva et al. in patients with haematological malignancies diagnosed with COVID-19, concluded that these patient subgroups have high mortality rates despite newer treatment modalities [11]. However, in 2022, the European Conference on Infections in Leukaemia (ECIL-9) updated its recommendations, with antivirals as the mainstay of treatment; there have been concerns about reduced activity of the monoclonal antibodies with different variants of the SARS-CoV virus [12]. The evolution of newer variants of the SARS-CoV virus highlights the importance of further research in this vulnerable patient group.

## Conclusions

This case highlights the complexities of managing COVID-19 in immunosuppressed patients, particularly those with haematological malignancies such as follicular lymphoma who are at an increased risk of prolonged and severe disease due to both their underlying condition and the immunosuppressive therapies they receive.

A multi-disciplinary team approach proved critical to refine the differential diagnosis and guide treatment. Adjusting the treatment to include monoclonal antibodies (sotrovimab), additional antivirals (Paxlovid, remdesivir), and IVIGs was vital to achieving viral clearance and clinical improvement, exemplifying the value of personalised treatment strategies. Furthermore, the role of cycle threshold (Ct) values in this case emphasised their utility in gauging viral load, allowing clinicians to assess the severity of the infection. While Ct values have limitations in broader population studies, in individual cases like this, they can offer valuable insights into disease activity and guide treatment decisions.
